# Employment status 1 year after out-of-hospital cardiac arrest in comatose patients treated with therapeutic hypothermia

**DOI:** 10.1186/cc10895

**Published:** 2012-03-20

**Authors:** K Kragholm, M Skovmoeller, AL Christensen, K Fonager, HH Tilsted, H Kirkegaard, I De Haas, BS Rasmussen

**Affiliations:** 1 Cardiovascular Research Center, Aalborg, Denmark; 2 Aarhus University Hospital, Aalborg, Denmark; 3 Aarhus University Hospital, Skejby, Aarhus, Denmark

## Introduction

Therapeutic-induced mild hypothermia (TIMH) with a core temperature of 32 to 34°C for 12 to 24 hours for comatose survivors of out-of-hospital cardiac arrest (OHCA) with ventricular fibrillation or tachycardia has improved survival and neurologic outcome [1,2]. The aim of this study was to evaluate the incidence of patients returning to work 1 year after survival of OHCA treated with TIMH.

## Methods

From 30 June 2004 to 30 June 2009, OHCA patients between 18 and 65 years of age treated with TIMH were identified by the Danish National Patient Registry and intensive unit registrations. Data were collected from ambulance and hospital records. Employment status was registered prior to and 1 year after OHCA from the Danish Ministry of Employment and Welfare database, using five work categories (WC): WC 1, working full-time and independent of any social welfare; WC 2, unemployed but able to work; WC 3, on sick leave and receiving social welfare; WC 4, substantially reduced ability to work: and WC 5, on early retirement.

## Results

One hundred and thirty-three patients were identified. Forty eight patients were excluded from the final analysis, of which 29 patients were not able to work at baseline (WC 3 to 5), 14 patients in WC 1 to 2 at baseline died in hospital, three patients died after hospital discharge and two patients had turned 65 years of age at follow-up and went on regular retirement. A total of 85 patients in WC 1 to 2 at baseline were included in the final analysis, of which 55 (64.7%) of these initially comatose patients with OHCA treated with TIMH had returned to work 1 year after OHCA.

## Conclusion

Approximately two-thirds of the survivors belonging to WC 1 to 2 at baseline have returned to work at 1 year follow-up after OHCA treated with TIMH. A larger study is needed to confirm these results and to determine predictors of returning to work in comatose patients after OHCA treated with TIMH.

## References

[B1] Bernard SA Gray TW Buist MD Jones BM Silvester W Gutteridge G Smith K Treatment of comatose survivors of out of hospital cardiac arrest with induced hypothermia N Engl J Med 2002 346 557 563 10.1056/NEJMoa003289 11856794

[B2] Hypothemia After Cardiac Arrest Study Group Mild therapeutic hypothermia to improve the outcome after cardiac arrest N Engl J Med 2002 346 549 556 11856793 10.1056/NEJMoa012689

